# *cor1* Gene: A Suitable Marker for Identification of Opium Poppy (*Papaver somniferum* L.)

**DOI:** 10.3390/foods13101432

**Published:** 2024-05-07

**Authors:** Eliška Čermáková, Pavel Svoboda, Jaroslava Ovesná, Jakub Vašek, Kateřina Demnerová, Kamila Zdeňková

**Affiliations:** 1 Department of Biochemistry and Microbiology, Faculty of Food and Biochemical Technology, University of Chemistry and Technology, Technická 5, 166 28 Prague, Czech Republic; demnerok@vscht.cz (K.D.); zdenkovk@vscht.cz (K.Z.); 2 Department of Chemistry, Biochemistry and Food Microbiology, Food Research Institute Prague, Radiová 1285/7, 102 31 Prague, Czech Republic; 3 Crop Research Institute, Drnovská 507/73, 161 06 Prague, Czech Republic; pavel.svoboda@vurv.cz (P.S.); ovesna@vurv.cz (J.O.); 4 Department of Genetics and Breeding, Faculty of Agrobiology, Food and Natural Resources, Czech University of Life Sciences Prague, Kamýcká 129, Suchdol, 165 00 Prague, Czech Republic; vasek@af.czu.cz

**Keywords:** *actin*, NADPH-dependent codeinone reductase, food fraud, opium poppy, PCR, species identification

## Abstract

This paper discusses the development of rapid, reliable, and accurate polymerase chain reaction (PCR) assays for detecting opium poppy (*Papaver somniferum* L.) in food. Endpoint, quantitative, and digital PCRs were compared based on the amplification of a newly developed DNA marker targeting the NADPH-dependent codeinone reductase (COR) gene. Designed assays were shown to be highly specific and sensitive in discriminating opium poppy from other plant species, even in heat-treated and food samples. Digital PCR was the most sensitive, with a detection limit of up to 5 copies, i.e., approximately 14 pg of target DNA per reaction. Quantitative and digital PCR further allowed the quantification of opium poppy in up to 1.5 ng and 42 pg (15 copies) of target DNA in a sample, respectively. In addition, two duplex PCRs have been developed for the simultaneous detection of opium poppy DNA and representatives of (i) the Papaveraceae family or (ii) the Plantae kingdom. Finally, all designed assays were successfully applied for analysis of 15 commercial foodstuffs; two were suspected of being adulterated. The study results have an important impact on addressing food fraud and ensuring the safety and authenticity of food products. Beyond food adulteration, the study may also have significant implications for forensics and law enforcement.

## 1. Introduction

The *Papaver* genus includes approximately 100 species distributed almost worldwide. The opium poppy (*Papaver somniferum* L.) is presumably the most important one [1,2,3,4,5]. *P. somniferum* varieties can be divided into three categories according to their use and properties: (i) industrial (i.e., opium poppies) grown for alkaloid production, (ii) culinary (i.e., oilseed poppies) grown for seed and oil production, and (iii) combined (i.e., dual), which can be used for both industrial and culinary purposes [1]. The chemical composition of poppy varieties changes due to growing conditions and geographic location.

The Czech Republic and Turkey are the most important growers of oilseed poppies in the world [6,7,8]. Only oilseeds obtained from cultivated varieties (cultivars) of *P. somniferum* that contain no more than 0.8% morphine alkaloids in poppy dry matter and whose total morphine alkaloid content on the surface of poppy seed (latex-derived) does not exceed 25 mg·kg^−1^ are authorised for food purposes [9]. Poppy seeds are mainly used as sprinkles and fillings in confectionery or bakery products [2,3,4,5,10]. It was found that seed processing such as roasting, grinding, or baking leads to a significant reduction in the morphine content. The amount of morphine in poppy seeds can also be changed by washing or soaking them in water [11,12]. This is used by fraudsters who mix or replace oilseed poppy seeds with low-quality seeds of industrial varieties, which do not have such good nutritional properties and may contain higher than permitted levels of alkaloid content.

The adulteration of poppy seeds and oil with other vegetable ingredients has also reduced the quality of poppy products [13,14]. There have been cases where poppy seeds were replaced by cheaper seeds that resembled them in colour and shape (e.g., amaranth, chia, or sesame). Poppy oil was, for example, adulterated with sunflower oil to increase the seller’s profits [15,16]. In some countries, the common poppy (*Papaver rhoeas*) is also used in food; its seeds are easily confused with the opium poppy. All these ways of adulterating poppies can endanger the health of the consumer.

Therefore, highly specific and sensitive methods for the analysis of poppy contents in food are necessary. Methods based on DNA analysis, which exploit the uniqueness of the DNA structure, are suitable for the detection of biological additives and the determination of poppy authenticity [17,18,19,20,21]. The high stability of DNA enables the detection of poppy even in processed products.

Nowadays, polymerase chain reaction (PCR) analysis is widely used for its simple analysis setup, available instruments, software, as well as chemicals, cost-effectiveness, and validation of results. Three types of PCR are distinguished: (i) conventional (end-point) PCR, (ii) quantitative PCR with fluorescent detection in real-time (qPCR), and (iii) digital PCR (dPCR). All three generations of PCR can be used for qualitative analyses; qPCR and dPCR can also be used for quantitative analyses. Compared to previous types of PCR, dPCR allows the analysis of rare target sequences in the DNA background and more accurate quantification without a calibration curve. 

This work deals with PCR-based methods for laboratory testing of poppy. A new genetic marker suitable for the detection of poppy DNA was developed. The gene encoding a key enzyme of the biosynthetic pathway of benzylisoquinoline alkaloids, the NADPH-dependent codeinone reductase (COR, EC 1.1.1.247) [22,23,24,25], was used as a target. Its position in biosynthetic pathways, together with its chromosomal localisation, as well as its presence in the cytosol of laticifers, leads us to study its potential for poppy DNA authentication. 

## 2. Materials and Methods

### 2.1. Samples

In this work, samples belonging to 25 plant and 4 animal species commonly used in food production were analysed (Table 1). At least 3 samples per species were used; opium poppy included 41 samples of 9 different cultivars (Aplaus, Bergamon, Buddha, Major, Maraton, Opal, Opex, Peony, and Postomi). Plant samples were provided by the Central Institute for Supervising and Testing in Agriculture (CISTA), Czech University of Life Sciences Prague (CZU Prague), and Crop Research Institute (CRI).

In addition, DNA from heat-treated opium poppy seeds (see Section 2.2) and 15 bakery products (poppy pies, strudel, and buns) purchased from the Czech market network were also included. All samples were homogenised by an analytical grinder IKA A10 (IKA-Werke, Staufen im Breisgau, Germany), weighed out, and stored at −20 °C until analysis.

### 2.2. Model Samples

In food, poppy seeds are usually thermally or mechanically treated. Therefore, model samples were prepared to verify the applicability of the method for this kind of sample. *P. somniferum* seeds were cooked and baked as described in Table 2. Analysis of all of these samples was carried out in the same way as with the plant samples listed in Table 1. 

### 2.3. DNA Isolation

DNA was extracted from seeds, leaves, stems, and/or poppyheads of the plant; animal DNA was obtained from muscles. DNA was isolated from 200 mg of homogenised samples by the CTAB method according to ČSN EN ISO 21571 (2005) [26] with the lysis step at 65 °C prolonged to 12 h. The quality of the isolated DNA was verified by horizontal agarose electrophoresis (1% agarose gel) stained by Midori Green Advance (Elisabeth Pharmacon, Croydon, UK). DNA concentration and purity were determined spectrophotometrically by a NanoPhotometer (Implen GmbH, Munich, Germany). DNA amplifiability was verified with Act-L primers (Table 3) and expressed as qPCR amplification efficiency according to Hougs, Gatto [27].

A comparison of two lysis periods (1 h × overnight/12 h incubation) was performed. In the end, the longer time (12 h) was chosen because visually, there was better decomposition of the dry poppyheads and seed samples, and it also proved to be practical when more samples needed to be processed at the same time. However, based on the spectrophotometric data, no significant difference in DNA purity and/or concentration was found as a result of different incubation times. Therefore, a 1 h lysis can also be used. The DNA was stored at +4 °C for a maximum of two weeks; long-term storage was performed at −20 °C.

### 2.4. In Silico Analysis of Actin and cor Genes

*In silico* analysis of *actin* and *cor* genes was performed based on nucleotide sequences available in the NCBI (National Center for Biotechnology Information; www.ncbi.nlm.nih.gov, accessed on 6 December 2022) or the ENA (European Nucleotide Archive; www.ebi.ac.uk/ena, accessed on 6 December 2022) databases. Nucleotide sequences of both genes were extracted from a *P. somniferum* reference genome (genome assembly ASM357369v1) and aligned using MEGA 11 software [28]. A consensus sequence was created for each gene, and highly similar sequences were searched in the Papaveraceae family to find a unique position of primers and probes for target DNA. The specificity of the designed oligonucleotides was further checked using Primer-BLAST (NCBI) against the Plantae kingdom. The nucleotide sequence of expected amplicons was analysed by blastn suite (NCBI) and Sequence Similarity Search (ENA) to search for possible false positive results and mismatches against the reference.

### 2.5. Primers and Probes

The primers and probes (East Port Prague, Czech Republic) used in this study are listed in Table 3. To verify the amplifiability of plant DNA, the universal primer pair for plant species, Act-L [29,30], was used. A second forward primer and probe for the *actin* gene, Actin-Z, were designed in this study to verify the presence of the Papaver species; COR1 was designed to amplify the DNA of *P. somniferum* only. Primers for myostatin were used as a positive control for animal DNA amplification.

**Table 3:** Sequence of oligo used in this work.

Marker	Oligonucleotide ^†^	Sequence (5′-3′)	Size [bp]	Reference
COR1	COR1_F	CCTTGTATAAATATCCCCGGA	207	This work
	COR1_R	TCTGATTATGCCCTTATTCAAC		
	COR1_P	FAM-AGTTGTTTCCATTTTTGGAGT CAAGTTGAGACA-BHQ-1		
Actin	Act-L_F	CAAGCAGCATGAAGATCAAGGT	~103	[29,30]
	Act-L_R	CACATCTGTTGGAAAGTGCTGAG		
	Act-L_P	HEX-CCTCCAATCCAGACACTGTA CTTYCTCTC-BHQ-1		
	Actin-Z_F	CCCTGGAATTGCTGATAGGATGA	150 ^‡^	This work
	Actin-Z_P	HEX-ATCACAGCTCTTGCACCAAGCAG CATGAAG-BHQ-1		
Myostatin	MY_F	TTGTGCAAATCCTGAGACTCAT	97	[31]
	MY_R	ATACCAGTGCCTGGGTTCAT		

Legend: COR—gene coding NADPH-dependent codeinone reductase; FAM—carboxyfluorescein; HEX—hexachlorofluorescein; BHQ—Black Hole Quencher^®^. ^†^ F, R and P in the oligo name indicate forward primer (F), reverse primer (R) and probe (P). ^‡^ Act-L_R is used as a reverse primer.

### 2.6. End-Point PCR

PCR amplification was conducted in a volume of 25 µL containing 2.5 mM MgCl_2_, 0.2 mM dNTP mix (Promega, Madison, WI, USA), primer mix (0.6 μM each), 100 ng template DNA, and 1.25 U GoTaq^®^G2 Flexi DNA polymerase (Promega, Madison, WI, USA). Amplifications were run using a Biometra T-Gradient thermocycler (Whatman Biometra, Göttingen, Germany) as follows: initial denaturation at 94 °C for 120 s, 35 cycles of denaturation at 94 °C for 30 s, annealing at 58 °C for 30 s, and extension at 72 °C for 30 s; final polymerization was 300 s at 72 °C. Visualization and detection of PCR products were carried out by 2.5% agarose gel stained by Midori Green Advance.

PCR controls have been included in each reaction; the positive control contained 100 ng of target DNA, whereas the no-template control was nuclease-free water (instead of DNA).

### 2.7. Quantitative Real-Time PCR (qPCR)

The qPCR amplification was performed with both types of fluorescence detection, an intercalating dye, and a specific dual labelled hydrolysis probe (TaqMan^TM^). Two commercial kits were used for this purpose, namely HOT FIREPol^®^ EvaGreen^®^ qPCR Supermix (Solis BioDyne, Tartu, Estonia) and GoTaq^®^ Probe qPCR Master Mix (Promega, Madison, WI, USA). Both mastermixes were prepared following the manufacturer’s instructions. The total reaction volume was 20 µL, and the amount of DNA added to the reaction was 100 ng in both cases.

For HOT FIREPol^®^ EvaGreen^®^ qPCR Supermix, the concentration of primers was 0.2 μM, and amplification was as follows: 95 °C for 720 s, followed by 35 cycles of 95 °C for 15 s, 58 °C for 20 s, and 72 °C for 30 s.

For GoTaq^®^ Probe qPCR Master Mix, the final concentration of primers and probe was 0.63 μM and 0.25 μM, respectively. Initial denaturation was carried out at 94 °C for 120 s, followed by 35 cycles of denaturation at 94 °C for 30 s, annealing at 58 °C for 30 s, and extension at 72 °C for 30 s. The final polymerization was 300 s at 72 °C.

All amplifications were performed on the ABI7500 thermocycler (Applied Biosystems, Waltham, MA, USA) and/or QuantStudio 5 (Applied Biosystems, Waltham, MA, USA). Data were analysed in 7500 Software v2.3 and Design and Analysis software v2.6.0 (Applied Biosystems, Waltham, MA, USA). A sample with a threshold cycle (Ct) higher than 30 was considered negative.

### 2.8. Droplet Digital PCR (ddPCR)

The ddPCR reaction mixture was prepared with 2× ddPCR^TM^ Supermix for Probes (no dUTP) or 2× QX200 EvaGreen ddPCR^TM^ Supermix following the instructions from Bio-Rad (Hercules, CA, USA). The final concentrations of primers were 0.1 μM when the EvaGreen supermix was used and 0.7 μM with Supermix for probes. The final concentration of the probe was 0.25 μM. The amount of DNA added to the reaction was 25 ng. After droplet generation, the ddPCR was carried out in a volume of 40 μL. The conditions of ddPCR followed the manufacturer’s instructions with one modification: the addition of an extension step (72 °C, 30 s) after annealing (at 58 °C). As recommended, 40 amplification cycles were used. Results were analysed by the QX200 droplet reader and QuantaSoft version 1.7.4 (Bio-Rad, Hercules, CA, USA). Samples with a copy number of less than 5 were considered negative.

### 2.9. Multiplex PCR

In this work, multiplex design was also tested for all types of PCR. Multiplex reaction mixtures differed from the singlet reaction only in the addition of oligonucleotides instead of the corresponding volume of nuclease-free water; the concentration of other substances was unchanged. Also, the thermal cycling conditions remained the same.

Each PCR type had a different combination and/or concentration of primers. In the case of end-point PCR, COR1 primers can be combined with both Actin-L and Actin-Z; 0.6 μM of each primer was used. The other method, qPCR, is set up depending on the type of signal detection, i.e., whether intercalating dye or a probe with fluorescent dye is used. In the intercalating dye arrangement, it is appropriate to combine only the COR1 primers with Actin-Z; the concentration of each primer was 0.2 μM. COR1 primers can be paired with both Actin-Z and Actin-L when using probes. The concentration of each primer was 0.6 μM and the probe concentration was 0.25 μM. For the ddPCR, the duplex response was tested only in the probe arrangement. COR1 oligonucleotides were tested in combination with both Actin-L and Actin-Z. The concentration of each primer was 0.7 μM, and the concentration of each probe was 0.25 μM.

### 2.10. Validation of Designed PCR Assays

The analytical characteristics of PCRs designed for the authentication of opium poppy have been determined following Hougs, Gatto [27] and the Codex Alimentarius guidelines (CAC/GL 74-2010) [32].

#### 2.10.1. Specificity

Known samples of opium poppy and non-target species commonly used in food (Table 1) were used to evaluate the property of PCR methods to respond exclusively to the target DNA. DNA from all tested samples was analysed at least three times, each time in duplicate, using the designed primer pairs.

#### 2.10.2. Sensitivity

An experimental evaluation of the sensitivity of the PCR assay was performed to establish the range of the method using different concentrations of opium poppy DNA. For analysis, DNA from known samples of opium poppy seeds was diluted serially at a 1:4 ratio six times; the initial amount was 100 ng. The absolute limit of detection (LOD) was defined as the lowest concentration of target DNA in the reaction that could be detected in at least 95% of the cases (samples). The limit of quantification (LOQ) was determined as the lowest concentration of target DNA in a reaction that can be reliably quantified with an acceptable level of precision and accuracy (repeatability 98%). For this purpose, DNA from known samples of opium poppy seeds was measured at least 10 times for each dilution level.

## 3. Results

The purpose of this study was to develop a rapid, robust, accurate, and cost-effective DNA-based methodology that would distinguish *P. somniferum* from other plant species. Firstly, it was necessary to select the appropriate DNA isolation protocol suitable for extraction from all parts of the plant and foodstuffs made with poppy seeds.

The CTAB method provided DNA isolates with concentration (range 25–700 ng·µL^−1^) and purity (A_260_/A_280_ in the range of 1.7–2.0) sufficient for further analyses of all tested samples.

### 3.1. Actin Gene as a Reference Marker and Control of DNA Amplification

Correct DNA quantification requires knowledge of the number of DNA sequences targeted by a taxon-specific PCR reference assay. It is also essential for harmonising measurement results and proper DNA quantification [33]. Based on these facts, an *in silico* analysis of the gene for actin, which is commonly used as a reference marker, was performed in this work. We searched for highly similar sequences (megablast) [34] to the PCR amplicon defined by Act-L primers to verify its suitability as a reference marker for poppy DNA analysis. The result of *in silico* analysis showed that more copies of the target sequences could be amplified, although it has been previously published that *actin* is a single-copy gene [29]. For example, sequences with more than 85 percent similarity were found at five different locations in the *Arabidopsis* reference genome (GCA_000211275.1). Similar results were also found in the genomes of *Brassica napus* (GCA_000686985.2), *Glycine max* (GCF_000004515.5), *Zea mays* B73 (GCF_000005005.2) and *Solanum tuberosum* (GCF_000226075.1). Two sequences with more than 90% similarity were found in the *Papaver somniferum* genome (assembly ASM357369v1) in chromosomes 9 and 11; several copies with lower similarity (80–89%) were found in the whole-genome shotgun sequence of the HN1 cultivar in chromosome 1 (4×), chromosome 6 (1×), chromosome 7 (2×), and chromosome 8 (1×). What is more important, the 3′ ends of the published primers were complementary to these sequences, and multiple loci could be amplified. Therefore, this target gene is inappropriate for quantification of plants’ haploid genome equivalent (HGE or C-value), since we do not know the exact number of copies in a given sample. On the other hand, the conservation of this sequence across the Plantae kingdom makes it a suitable reference gene to verify the ability to amplify DNA isolated from plants or food containing plant material.

**Figure 1:**
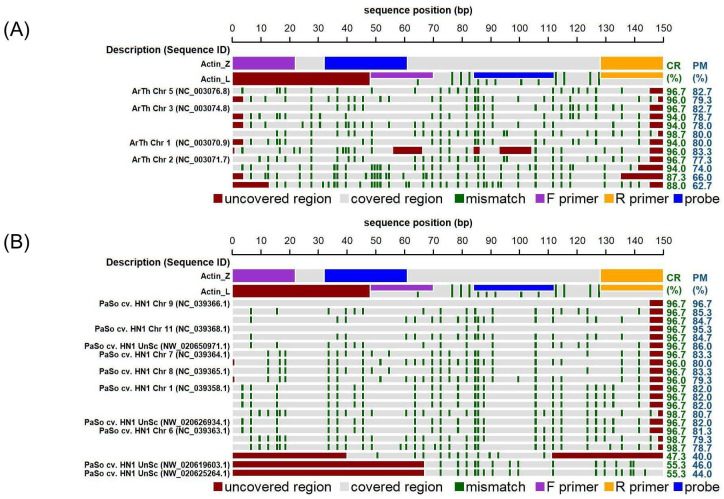
*In silico* analysis of actin oligonucleotides (Act-L and Act-Z) demonstrating significant alignments within the genomes of (**A**) *Arabidopsis thaliana* (Genome assembly TAIR10.1) and (**B**) *Papaver somniferum* (genome assembly ASM357369v1). Individual lanes in the diagram depict *actin* sequences (Act-L and Act-Z) along with the significant alignments produced through nucleotide BLAST (discontiguous megablast) for a 150 bp segment of the Act-Z sequence in the respective genome. Numbers within brackets indicate the sequence identifier. The positions of primers and probes corresponding to the *actin* sequences are detailed according to the legend provided below the plot. Dark green stripes emphasize mismatches relative to the original Act-Z sequence. The ‘Covered region’ (CR) and ‘Perfect match’ (PM) metrics specify the percentages of the Act-Z sequence aligned with, and perfectly matched to, the target sequence, respectively, while areas not aligned are marked in red. Abbreviations include “Chr” for chromosome, “UnSc” for unplaced scaffold, and “cv.” for cultivar.

To improve the comparison of PCR results across the poppy species, we designed new oligonucleotides (Actin-Z) complementary to the poppy *actin* gene. Also, in this case, more copies of the *actin* gene are expected; however, the number of copies is supposed to be more consistent within a family. Based on the *in silico* analysis, predicted amplicons of *P. somniferum* (ASM357369v1) originated from loci of chromosomes 9 and 11. An example of *in silico* analyses of Actin-L and Actin-Z primer annealing sites in the genomes of *P. somniferum* and *A. thaliana*, a model plant organism, is shown in Figure 1. For both targets (Act-L, Act-Z), alignments were also performed with the nucleotide database within higher plants (taxid: 3193) (Figure S1).

The results of the *in silico* analysis were further verified experimentally. Actin-L primers amplified all plant DNA samples. This confirmed the suitability of the isolates for further analysis. Actin-Z amplified poppy DNA in end-point PCR as expected; the amplicon length was approximately 150 bp for all tested species (Figure S2). In addition to poppy, Actin-Z also amplified amaranth, mustard, oats, wheat, rice, and potato in qPCR using a mastermix with EvaGreen dye. However, using the fluorescently labelled probe, Actin-Z showed high specificity for poppy DNA (Figure S3). This approach also allows a reliable estimation of the amount of poppy DNA presented in the sample. It was verified that the designed assays with the Actin-Z marker enable the quantification of opium poppy DNA up to a concentration of 1.5 ng in the reaction by qPCR and 42 pg (i.e., 15 copies) by the ddPCR platform. These assays can thus allow early detection of undeclared admixtures of poppy, which is an allergen, in food.

The amplifiability of animal DNA was verified with MY primers (Table 2) universal for mammals and poultry. All samples were successfully amplified.

### 3.2. cor Gene as a Marker for P. somniferum Discrimination

The gene coding NADPH-dependent codeinone reductase protein (COR) is a promising DNA marker for the identification of *P. somniferum*. *In silico* analysis of the *cor* gene showed high consistency within *P. somniferum* cultivars; similarity with other poppy species was less than 90% (compared to KY688197.1 accession number). In addition, the second intron of the *cor* allows us to distinguish this gene from the pseudogene thanks to the indels in this area in *P. somniferum.* Oligonucleotides have therefore been proposed for this region.

**Figure 2:**
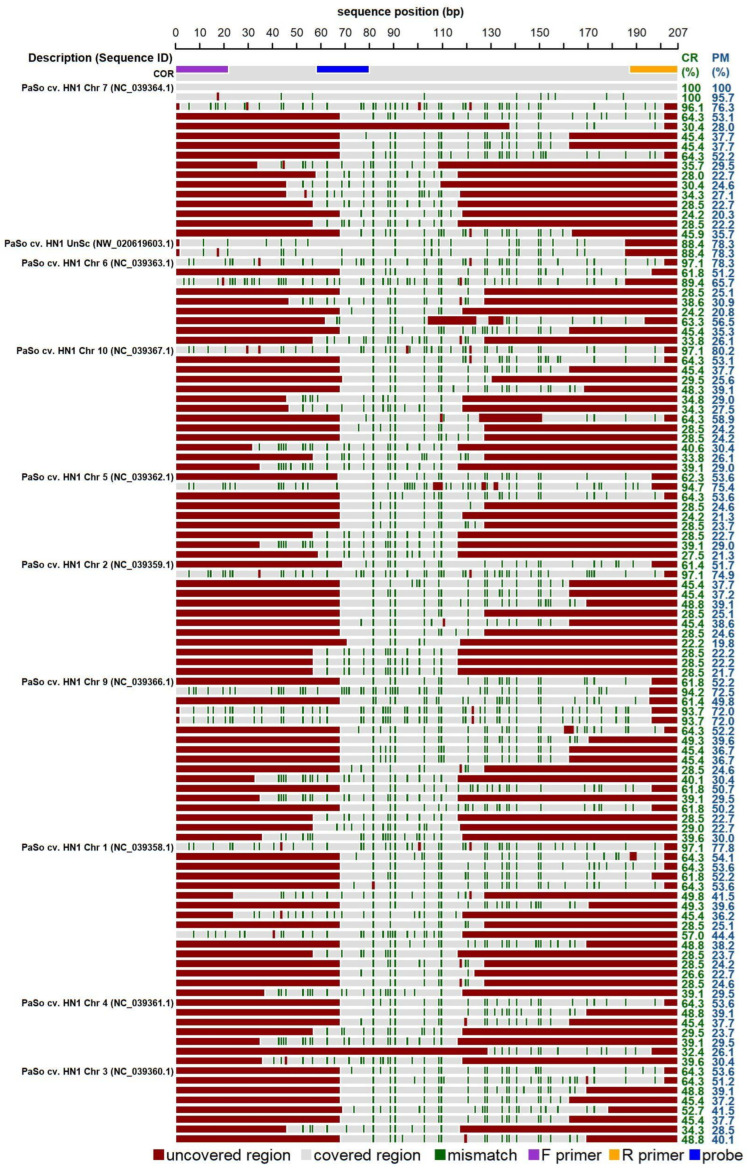
*In silico* analysis of codeinon reductase oligonucleotide (COR) demonstrating significant alignments within the genome of *Papaver somniferum* (genome assembly ASM357369v1). The individual lanes in the diagram depict COR sequence along with significant alignments generated within nucleotide BLAST (discontiguous megablast) for the genome of interest. Numbers within brackets indicate the sequence identifier. The positions of primers and probe corresponding to the *cor* sequences are detailed according to the legend provided below the plot. Dark green stripes emphasize mismatches relative to the original COR1 sequence. The “Covered region” (CR) and “Perfect match” (PM) metrics specify the percentages of the COR1 sequence aligned with, and perfectly matched to, the target sequence, respectively, while areas not aligned are marked in red. Abbreviations include “Chr” for chromosome, “UnSc” for unplaced scaffold, and “cv.” for cultivar.

The expected amplicon was searched in the reference genome of *P. somniferum* (genome assembly ASM357369v1). Sequences with more than 85 percent similarity (‘perfect match’) were found only at chromosome 7. Similar sequences with a percentage of “covered region” higher than 85% were found within the seven chromosomes and unplaced scaffold. However, these sequences contain a lot of mismatches, and for some of them, the area of the reverse primer is uncovered (Figure 2). *In silico* analysis was further performed for the available genomes of other poppy species (*P. armeniacum*, *P. atlanticum*, *P. bracteatum*, *P. californicum*, and *P. nudicaule*). The results of the analyses showed a low probability of their amplification by the proposed COR1 oligonucleotides.

Further, it was experimentally verified that the PCR with COR1 primers amplified only one of the seven tested poppy species—*P. somniferum*. The length of the amplicon (207 bp) remained the same regardless of the cultivar. However, the effect of the composition of the used reaction mixture on the specificity of the PCR assay was observed. For example, barley DNA was also detected on agarose gel when HOT FIREPol^®^ EvaGreen^®^ qPCR Supermix was used in the end-point PCR; no amplification was observed with GoTaq^®^ G2 Flexi DNA polymerase (see Figure S4). Different results may be caused by various processivities of the polymerases. Therefore, optimization of the reaction conditions when changing the mastermix is essential.

Weak DNA amplification (Ct in the range 30–35) of 7 non-poppy species was observed in qPCR using EvaGreen fluorescent detection, namely barley, maize, oilseed rape, pepper, rye, sorghum, and sunflower. Detection of mustard and wheat was on the “edge of positivity”, with an average Ct of 34.2 ± 4.4 and 31.8 ± 2.3, respectively (Table 4). Despite that, it was possible to safely distinguish opium poppy from other plant species based on the melting curve analysis (Figure S3). Nevertheless, the TaqMan^®^ probe was designed to achieve better specificity and improve the quantification limit of the qPCR assay. High specificity of the qPCR assay was demonstrated as only opium poppy DNA was amplified. Based on the results achieved by qPCR, only the assay using the COR1 probe was tested on the ddPCR platform. Optimized ddPCR assay was applied to all samples listed in Table 1. Weak amplification occurred in barley, pepper, and mustard samples. As less than 5 copies were detected, these samples were considered negative. The fluorescence emission at values between those of precisely negative and positive droplets could be caused by partial PCR inhibition in some droplets [35] or by droplet damage [36].

The results of COR1 primer specificity testing by end-point PCR, qPCR, and ddPCR are summarised in Table 4. In addition to the specificity test, the detection limit was also estimated, and the possibility of quantification using the proposed qPCR and ddPCR protocols was verified for both the culinary and dual cultivars of *P. somniferum*. The LOD was 98 pg for end-point PCR, 24 pg for qPCR, and 5 copies, i.e., approximately 14 pg, of target DNA in the reaction by ddPCR using the COR1 probe. The LOQ was set at a concentration of 1.5 ng in the reaction on the qPCR platform and 15 copies (app. 40 pg) by ddPCR.

### 3.3. Comparison of Three PCR Types

All the proposed PCR assays demonstrated the ability to detect opium poppy DNA. However, false-positive DNA amplification results may occur in the presence of plants from other families when using endpoint PCR or qPCR with fluorescence detection of intercalation dye. To increase specificity across families, we recommend the use of qPCR and/or ddPCR using a fluorescence probe. Both of these PCR arrangements also enable DNA quantification, which can be an advantage, especially when analysing food products and detecting impurities caused by other types of poppies. The advantages of ddPCR are a high tolerance to inhibitors, a lower detection limit, high sensitivity and accuracy, and the possibility of absolute quantification (without the need for a quantification standard). It also enables reliable quantification of a low concentration of the target segment in a high amount of accompanying nucleic acids. However, compared to qPCR, the disadvantage is more demanding optimization and a higher susceptibility to DNA quality. When using degraded DNA or sub-optimal annealing temperatures of primers, the rain effect can occur (Figure S5). The amplification results cannot be evaluated due to the inability to discriminate between positive and negative droplets. An essential prerequisite for the correct course of ddPCR is also the selection of an appropriate DNA concentration to ensure that the system is not completely saturated (the presence of negative droplets is necessary). End-point PCR and qPCR assays are more robust in this case.

Designed PCR assays were successfully used for heat-treated poppy seeds and commercial samples (see below). Only for ddPCR, the analysis of the results was more difficult for heat-treated poppies and commercial products because a rain effect appeared; however, the reactions were still evaluable. Since poppy seeds are often contained in a wide range of commercial food products in Central Europe, it is essential to provide consumers with information about allergens used as ingredients and/or presented as potential contaminants. For the analysis of food samples where there is a risk of DNA damage due to heat treatment of seeds or the ratio of poppy and other types of plants in the product is not known in advance, we recommend the use of qPCR.

### 3.4. Multiplexing of the PCR Assays

Duplex PCR using a combination of COR1 and Act-Z or Act-L primers was investigated. Multiplex reactions enable simultaneous detection of poppy DNA and other poppies or plants in the sample in one reaction, thereby reducing both the cost and time required for sample analysis. As far as we know, such a duplex has not been published yet.

**Figure 3:**
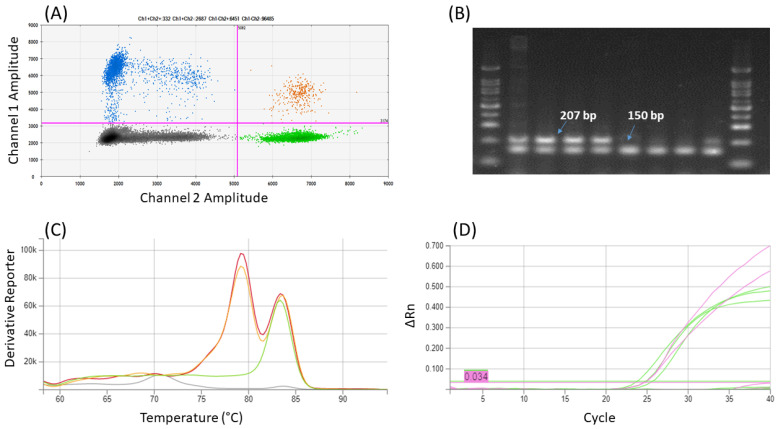
An example of results of duplex COR+Act-Z (**A**) ddPCR (2D plot): Four clusters of droplets were identified as single-positive for Channel 1—FAM (blue) and Channel 2—HEX (green), double-positive (orange) and double-negative (black); (**B**) Agarose gel after end-point PCR: Marker 100 bp, *P. somniferum* (Aplaus, Buddha, Maraton, Opal), *P. orientale*, *P. nudicaule*, *P. rhoeas*, *P. somniferum* (Postomi); (**C**) qPCR with EvaGreen (melting curves): *P. somniferum* in red and orange, *P. nudicaule* in green, NTC in grey color; (**D**) qPCR with probe: amplification curves with Act-Z (green—*P. nudicaule, P. rhoeas*) and COR (pink—*P. somniferum*) primers.

The specificity of the method was verified on selected plant species only. It was verified that a duplex of COR1 and Actin-Z primers with EvaGreen dye detection enables the differentiation of opium poppy DNA amplicons (COR1 primers) from other poppies (Act-Z) by melting curve analysis (Figure 3C); Act-L primers, which are used for verification of plant DNA amplifiability and detection of non-poppy admixtures, provided a melt peak for *P. somniferum* (Tm 79.3 ± 0.1 °C) too close to the COR1 amplicons (Tm 79.6 ± 0.1 °C) in duplex assay. It was also observed that in the case of the Act-L primers, there was a large shift in the melting temperature compared to the single reaction, when the Tm was 81.9 ± 0.3 °C. Compared to this, the assays with other primers are more robust: Tm of COR1 amplicons was 79.2 ± 0.1 °C in single reaction, ΔTm for Act-Z was 0.2 (Tm 83.3 ± 0.3 °C in single, 83.5 ± 0.1 °C duplex reaction). Although the Act-Z amplicon is shorter than COR1, its melting curve has a higher Tm. This is probably due to the higher abundance of GC bases in the *actin* (49%) than in the *cor* (33%) amplicon.

Both duplexes with probes performed well for *P. somniferum* (Aplaus, Buddha) authentication; the specificity of the designed assays was consistent with the appropriate single reactions in qPCR as well as ddPCR. The measured ΔCt between COR1 in a duplex with probe and appropriate qPCR single reaction was less than 0.5; the difference between Actin-Z and COR1 was 2.7 and 1.4, respectively. As we expected, in the case of *P. rhoeas*, flax, mustard, soya, and rice, the product was detected only with Act-L primers. Also, ddPCR gave satisfactory results. It was proven that the DNA concentration for Actin-Z is approximately two times (about 1.7–2) higher than for COR1.

### 3.5. Analysis of Heat-Treated Seeds and Real Food Samples

The applicability of the protocol for the analysis of bakery products was first verified on heat-treated poppy seeds. The treatment of the seeds was carried out as indicated in Table 2 and further underwent the same processing procedure (homogenization, DNA isolation, and amplification) as untreated seed samples. The results proved the applicability of the developed protocol for treated seeds, and it is therefore possible to predict good applicability for the analysis of food samples. Therefore, we analysed DNA from 15 food samples containing poppy with the Actin-Z and COR1 primers. Actin provided PCR products for all bakery products. However, in the case of the COR1 primers, DNA extracted from two pies was not amplified in qPCR (Table S1). These pies are thus suspected of being adulterated with various species of poppy and/or other plants. To confirm the obtained results, we further analysed the food products using ddPCR. In the case of samples suspected of adulteration, amplification with COR1 primers occurred, but there was insufficient separation of the droplets (rain effect). Therefore, it is not possible to reliably analyse the data and determine the amount of poppy. Since ddPCR provided a *cor* amplicon but qPCR did not, other poppy than opium poppy was probably mainly used in the filling of pies.

## 4. Discussion

DNA-based methods have good discriminatory power to identify biological admixtures in a wide range of foods. These methods exploit the uniqueness of each species’ DNA and the high stability of DNA, which allows the detection of adulterants even in processed products.

The extraction of nucleic acids is the first and essential step in the molecular-based analysis of food. Many factors affect the success of DNA isolation; for example, the plant matrix and the related variety of metabolites contained in the plant cell, as well as other undesirable substances (lipids, proteins, and others) [37,38,39]. There is no universal protocol for DNA extraction yet; the most appropriate extraction method should be chosen on a matrix basis. Thus, the amplification capability of the DNA isolated from a food sample needs to be verified because the DNA extract may contain PCR inhibitors [29,30,40,41,42]. For this purpose, universally amplifiable (conservative) DNA sequences of the *actin* gene were used. Moreover, their short length allowed DNA amplification even in heat-treated and complex samples. Experiments with oligonucleotides specifically designed for amplification of Papaveraceae (Actin-Z) and opium poppy (COR1) were performed after DNA amplification of the sample was verified by PCR amplification of plant *actin*.

The part of the *actin* gene defined by Actin-L primers is usually presented as a single-copy genomic marker that is detectable in (almost) the entire Plantae kingdom [29,43,44]. This predisposes it to be an ideal reference marker for both the detection and quantification of poppy DNA in a sample. However, *in silico* analysis performed in this work showed that more copies of the target sequences could be amplified in many plants. The 3′ ends of the actin primers were complementary to the multiple target sequences, so the PCR results would depend on the amplification conditions. Because the exact number of *actin* copies in a given plant is unknown, the Actin-L is inappropriate for quantification of the plants’ haploid genome equivalent (HGE or C-value). Despite the different copy numbers in plant genomes, this target sequence has the advantage of being sufficiently conserved across plant species. Therefore, it can be used as a reference gene to verify the ability to amplify genomic DNA isolated from plants or food containing plant material. To better compare PCR results across the tested poppy species, we recommended Actin-Z oligonucleotides that amplify mainly representatives of the Papaveraceae family.

A comparison of the amplification results of Actin-L and Actin-Z primers can demonstrate the adulteration of opium poppy products. Actin-L can reliably prove adulteration of poppy by other plants, e.g., amaranth, chia, or sunflower. On the other hand, Actin-Z can confirm the undeclared addition of poppy to the sample. Primers amplifying *cor* were designed for the determination and quantification of opium poppy DNA only. DNA sequences of Papaveraceae COR genes and pseudogenes available from the NCBI and ENA databases contain highly homologous coding regions. Thanks to minimal selection during the evolutionary process, the sequences of introns are more diverse and, therefore, suitable for opium poppy identification.

Lee, Hwang [45] published two primer pairs complementary to the *P. somniferum cor* gene, but neither was specific to *P. somniferum* only; *P. bracteatum*, *P. nudicaule*, *P. orientale,* and *P. rhoeas* were also amplified. PCR analysis of poppy using genes involved in the biosynthesis of benzylisoquinoline alkaloids (BIAs), including the *cor*, was also published by Choe, Lee [46]. The length of the amplicons was not mentioned in the publication. By *in silico* analysis of the NCBI database, we found that different sizes of amplicons could be observed in *P. somniferum* (length 915, 966, or 1644 bp), *P. rhoeas* (1316 bp), *P. orientale* (1632 bp), *P. bracteatum* (1632 bp), or *P. nudicale* (1345 bp). The various lengths of the products in individual poppy species would allow them to be distinguished; however, detecting such long products in heat- or chemically treated seeds could be problematic due to possible DNA degradation. For this reason, we consider our marker to be very useful as it provides correct identification of *P. somniferum*, even in processed seeds and food products. Based on the results obtained, two products were suspected of adulteration. Actin-Z primers provided the product, confirming the presence of poppy DNA. However, there was no amplification by qPCR with COR primers.

PCR detection of trace amounts of potentially allergenic sunflower and poppy seeds in commercial food products was also addressed by López-Calleja, de la Cruz [10]. The traces of poppy were found in both types of products tested, i.e., with or without the declaration of poppy or its traces on the label, using a duplex qPCR targeting a small subunit of the ribosomal RNA gene. According to *in silico* analysis of NCBI databases, oligonucleotides targeting a poppy are also complementary to other members of the *Papaveraceae* family, including *P. orientale*, *P. bracteatum*, *P. pseudoorientale,* and *Meconopsis cambrica*.

Another duplex qPCR, based on internal transcribed spacer (ITS1) amplification, has been reported for the specific identification of sunflower and *Papaver rhoeas* [10]. Multiplex analysis for opium poppy identification was also published Vašek, Čílová [47], Vašek, Čílová [48], and Svoboda, Vašek [49]. In these studies, identification was performed by simple sequence repeat (SSR) markers. The published markers have made it possible to distinguish the different poppy cultivars from each other, which is particularly useful for the possible monitoring of food adulteration. However, the disadvantage is the laboriousness and necessity of expensive laboratory equipment, such as capillary electrophoresis. Therefore, in the present work, we focused on the development of methodologies through all types of PCR analysis so that the proposed protocol can be used in the widest possible range of laboratories.

In our point of view, endpoint PCR or qPCR is more suitable for amplifiability verification and amplicon detection than ddPCR due to its easier and less time-consuming sample preparation, as well as its economic aspects. Moreover, the rain effect in ddPCR was abundant in food analysis. Although the ddPCR method has been proven in the past to be suitable for the authentication of commodities of animal and plant origin [50,51,52,53], it is still one of the least used types of PCR. On the other hand, ddPCR is less sensitive to the presence of inhibitors, quantification is highly accurate, and it does not require the preparation of a calibration curve. Thus, the use of ddPCR may be useful in monitoring trace amounts of poppy DNA in a sample. In addition, designed PCRs can be useful for monitoring the effectiveness of cleaning processes in the production units of the food industry.

## 5. Conclusions

In this study, we have successfully developed rapid, precise, reliable, and accurate quantitative PCR assays based on the amplification of the *cor* gene. The PCR assay using primers and a probe designed to anneal within the second intron of the *cor* gene has demonstrated the capability to distinguish *Papaver somniferum* from other *Papaveraceae,* thereby enabling the verification of the botanical origin and the detection of adulteration (botanical impurities) of food, even for heat-treated products. As far as we know, this study represents the first to determine the biological origin of *Papaver somniferum* by all three generations of PCR (endpoint, qPCR, and ddPCR). This should allow laboratories to choose an appropriate method based on their specific equipment, staff expertise, or time and financial constraints. The optimized methods have been effectively applied to the analysis of 15 food samples from the market network in the Czech Republic, leading to the identification of two bakery products suspected of adulteration due to the presence of a plant other than *Papaver somniferum* in their fillings. Designed PCR assays hold significant potential for verifying the authenticity of food products as well as detecting the presence of possible allergens and/or illegal plants.

## Figures and Tables

**Table 1:** List of taxa used in this study.

Family	Genus	Species	Common Name	Source
Papaveraceae	*Argemone*	*A. mexicana*	Mexican poppy	Seeds, leaves
	*Papaver*	*P. bracteatum*	Iranian poppy, Persian poppy	Leaves
		*P. commutatum*	Caucasian scarlet poppy	Leaves
		*P. glaucum*	Tulip poppy	Seeds, leaves
		*P. nudicaule*	Iceland poppy	Seeds, leaves
		*P. orientale*	Oriental poppy	Seeds, leaves
		*P. rhoeas*	Common poppy	Seeds, leaves
		*P. somniferum*	Opium poppy	Seeds, leaves, stems, poppyheads
Amaranthaceae	*Amaranthus*	(not specified)	Amaranth	Seeds
Asteraceae	*Helianthus*	*H. annuus*	Sunflower	Seeds
Brassicaceae	*Brassica*	*B. napus*	Rape	Leaves
	*Sinapsis*	*S. alba*	White mustard	Leaves
Fabaceae	*Glycine*	*G. max*	Soya-bean	Leaves
	*Medicago*	*M. sativa*	Alfalfa	Seeds, leaves
Linaceae	*Linum*	*L. usitatissimum*	Common flax	Seeds
Poaceae	*Avena*	*A. sativa*	Common oat	Grain, leaves
	*Hordeum*	*H. vulgare*	Six-rowed barley	Grain, leaves
	*Oryza*	*O. sativa*	Burgundy rice	Leaves
	*Secale*	*S. cereale*	Cereal rye	Grain, leaves
	*Sorghum*	*S. bicolor*	Great Millet, Sorghum	Seeds, leaves
	*Triticum*	*T. aestivum*	Bread wheat	Grain, leaves
	*Zea*	*Z. mays*	Maize	Grain, leaves
Solanaceae	*Capsicum*	*C. annuum*	Sweet Pepper	Leaves, pod
	*Solanum*	*S. tuberosum*	Potato	Tissue
		*S. lycopersicum*	Tomato	Leaves
Bovidae	*Bos*	*B. taurus*	Cattle	Tissue
Equidae	*Equus*	*E. caballus*	Horse	Tissue
Phasianidae	*Gallus*	*G. gallus*	Chicken	Tissue
Suidae	*Sus*	*S. scrofa*	Pig	Tissue

**Table 2:** The heat treatment of poppy seeds.

Treatment Conditions	Type of Treatment		Poppy Seeds
80 °C, 30 min	Baking		Whole
			Ground
100 °C, 2 min	Cooking	in water	Ground
		in milk with the addition of sugar	
180 °C, 30 min	Baking		Seeds after cooking in water
			Seeds after cooking in milk
			Ground (without previous cooking)
			Whole (without previous cooking)
Dried at room temperature; without heat treatment			Ground

**Table 4:** An overview of DNA amplification results.

Group	Sample		DNA Amplification Control ^†^	Amplification of *cor* Gene ^‡^		
			PCR (All Tested Platforms)	PCR	qPCR (EvaGreen/Probe)	ddPCR (Probe)
**Papaveraceae**	Opium poppy	Aplaus	+	+	+	+
		Bergamon	+	+	+	+
		Buddha	+	+	+	+
		Major	+	+	+	+
		Maraton	+	+	+	+
		Opal	+	+	+	+
		Opex	+	+	+	+
		Peony	+	+	+	+
		Postomi	+	+	+	+
	Caucasian scarlet poppy		+	−	−	−
	Common poppy		+	−	−	−
	Iceland poppy		+	−	−	−
	Iranian or Persian Poppy		+	−	−	−
	Mexican Poppy		+	−	−	−
	Oriental Poppy		+	−	−	−
	Tulip poppy		+	−	−	−
**Other plant species**	Alfalfa		+	−	−	−
	Amaranth		+	−	−	−
	Barley		+	−	−	−/+
	Flax		+	−	−	−
	Maize		+	−	−	−
	Mustard		+	−	+/−	−/+
	Oat		+	−	−	−
	Oilseed rape		+	−	−	−
	Pepper		+	−	+/−	−
	Potato		+	−	+/−	−
	Rice		+	−	−	−
	Rye		+	−	−	−
	Sorghum		+	−	−	−
	Soya		+	−		−
	Sunflower		+	−	−	−
	Tomato		+	−	−	−
	Wheat		+	−	+/−	−
**Animal species**	Cattle		+	−	−	−
	Horse		+	−	N	−
	Chicken		+	−	−	−
	Pig		+	−	−	−

^†^ Amplification was verified by PCR with Actin-L, Actin-Z or MY primer pairs. ^‡^ Symbol “+” indicates positive detection, “−” negative detection, and “−/+” is around LOD (both positive and negative results have been achieved) of PCR method. The cut-off Ct was set at 30; for ddPCR the LOD was set at 5 copies in the reaction.

## Data Availability

Data available on request. The data are not publicly available due to privacy restrictions.

## Supplementary Materials

The following supporting information can be downloaded at: https://www.mdpi.com/article/10.3390/foods13101432/s1, Figure S1: The example of *in silico* analysis of actin oligonucleotides, Act-L and Act-Z, showing their significant alignments against a nucleotide database and within higher plants (taxid: 3193). It features lanes that represent the Act-L and Act-Z sequences, highlighting their alignments identified by nucleotide BLAST (discontiguous megablast) for a specific 150 bp segment of the Act-Z sequence in the targeted organisms. Sequence identifiers are noted within brackets. The diagram also details the positions of primers and probes for the actin sequences, as outlined in the legend below the plot. Mismatches compared to the original Act-Z sequence are marked with dark green stripes, while the ‘Covered region’ and ‘Perfect match’ values indicate the percentages of the Act-Z sequence that align with the target sequence, with and without mismatches, respectively. Areas without alignment are highlighted in red. The abbreviations used include ‘Chr’ for chromosome, ‘UnSc’ for unplaced scaffold, and ‘cv.’ for cultivar. Figure S2: An example of Actin-Z and COR1 primers specificity test in end-point PCR aimed at amplification of poppy DNA. Figure S3: Examples of the results obtained with COR and Actin-Z primers when qPCR with EvaGreen^®^ (A) and TaqMan^®^ probe (B) detection was used. Figure S4: An example of specificity test with GoTaq^®^ G2 Flexi DNA polymerase (Promega, Madison, WI, USA) and HOT FIREPol^®^ EvaGreen^®^ qPCR Supermix (Solis BioDyne, Tartu, Estonia) in end-point PCR using COR1 primers. Figure S5: Optimization of ddPCR assay with COR1 primers. Table S1: List of analysed samples from the Czech market network.
