# Supplementary material for: cor1 Gene: A Suitable Marker for Identification of Opium Poppy (Papaver somniferum L.)
Source: Foods. 2024 May 7;13(10):1432. doi: 10.3390/foods13101432 (PMC11120590; doi:10.3390/foods13101432)
Supplement: Supplementary file 1 [file foods-13-01432-s001.zip › foods-2963943-supplementary.pdf]

Supplementary data:

Figure S1: The example of *in silico* analysis of actin oligonucleotides, Act-L and Act-Z, showing their significant alignments against a nucleotide database and within higher plants (taxid: 3193). It features lanes that represent the Act-L and Act-Z sequences, highlighting their alignments identified by nucleotide BLAST (discontiguous megablast) for a specific 150 bp segment of the Act-Z sequence in the targeted organisms. Sequence identifiers are noted within brackets. The diagram also details the positions of primers and probes for the actin sequences, as outlined in the legend below the plot. Mismatches compared to the original Act-Z sequence are marked with dark green stripes, while the 'Covered region' and 'Perfect match' values indicate the percentages of the Act-Z sequence that align with the target sequence, with and without mismatches, respectively. Areas without alignment are highlighted in red. The abbreviations used include 'Chr' for chromosome, 'UnSc' for unplaced scaffold, and 'cv.' for cultivar.

Figure S2: An example of Actin-Z and COR primers specificity test in end-point PCR aimed at amplification of poppy DNA.

Figure S3: Examples of the results obtained with COR and Actin-Z primers when qPCR with EvaGreen® (A) and TaqMan® probe (B) detection was used.

Figure S4: An example of specificity test with GoTaq® G2 Flexi DNA polymerase (Promega, USA) and HOT FIREPol® EvaGreen® qPCR Supermix (Solis BioDyne, Estonia) in end-point PCR.

Figure S5: Optimization of ddPCR assay with COR primers.

Table S1: List of analysed samples from the Czech market network.

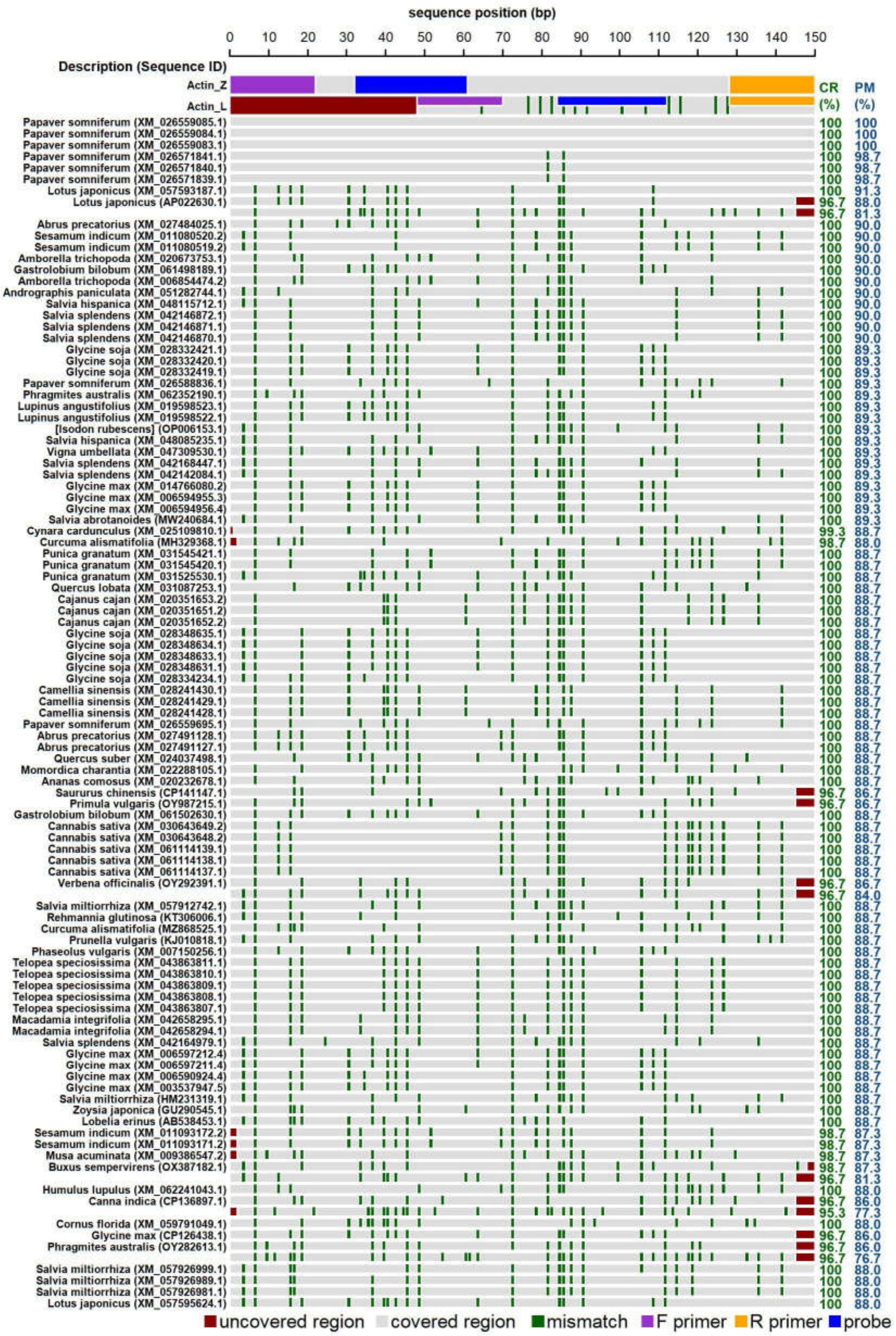

Figure S1: This figure illustrates the *in silico* analysis of actin oligonucleotides, Act-L and Act-Z, showing their significant alignments against a nucleotide database and within higher plants (taxid: 3193). It features lanes that represent the Act-L and Act-Z sequences, highlighting their alignments identified by nucleotide BLAST (discontiguous megablast) for a specific 150 bp segment of the Act-Z sequence in the targeted organisms. Sequence identifiers are noted within brackets. The diagram also details the positions of primers and probes for the *actin* sequences,

as outlined in the legend below the plot. Mismatches compared to the original Act-Z sequence are marked with dark green stripes, while the 'Covered region' and 'Perfect match' values indicate the percentages of the Act-Z sequence that align with the target sequence, with and without mismatches, respectively. Areas without alignment are highlighted in red. The abbreviations used include 'Chr' for chromosome, 'UnSc' for unplaced scaffold, and 'cv.' for cultivar.

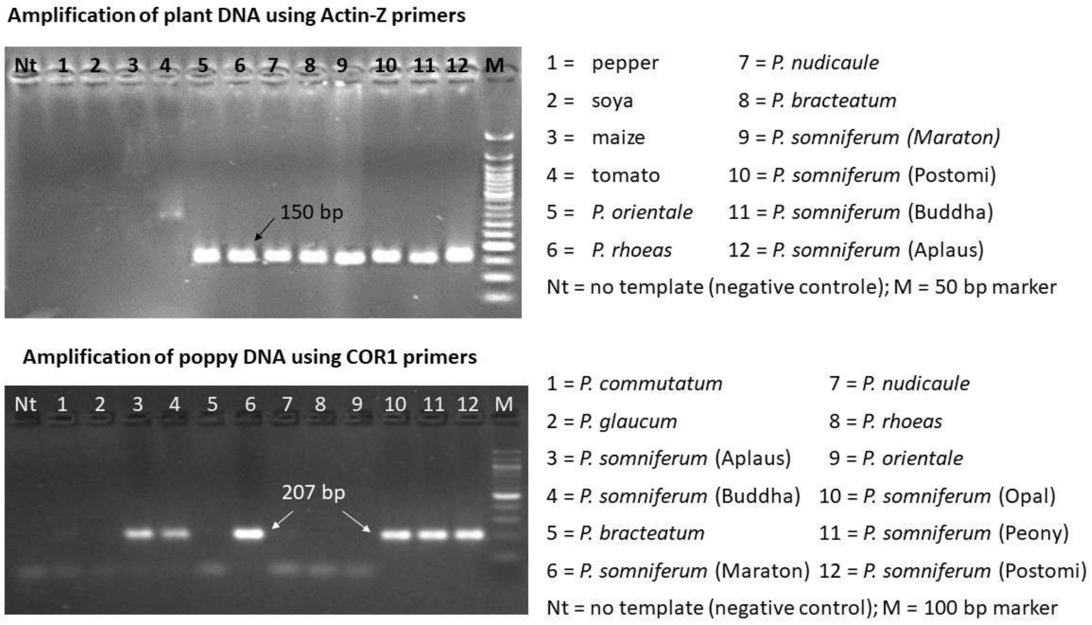

Figure S2: An example of Actin-Z and COR primers specificity test in end-point PCR aimed at amplification of poppy DNA.

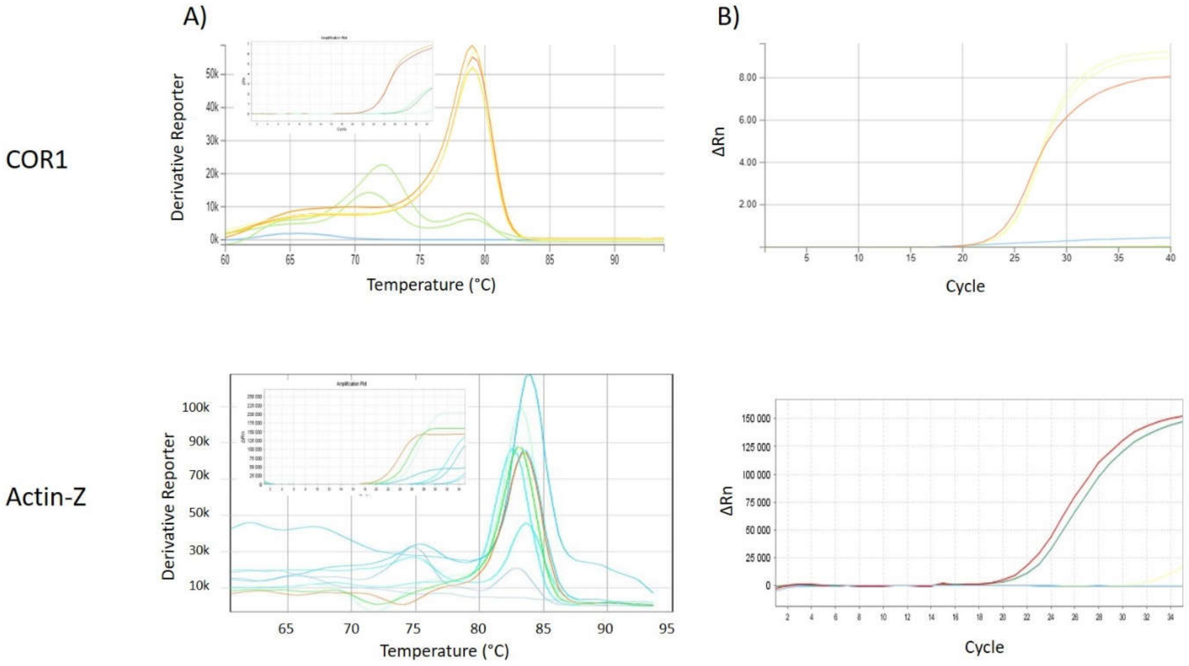

Figure S3: Examples of the results obtained with COR and Actin-Z primers when qPCR with EvaGreen® (A) and TaqMan® probe (B) detection was used. The red and yellow colour of melting curves = opium poppy DNA (different cultivars), green melting curves = other plant species DNA, blue line = no template and soya DNA.

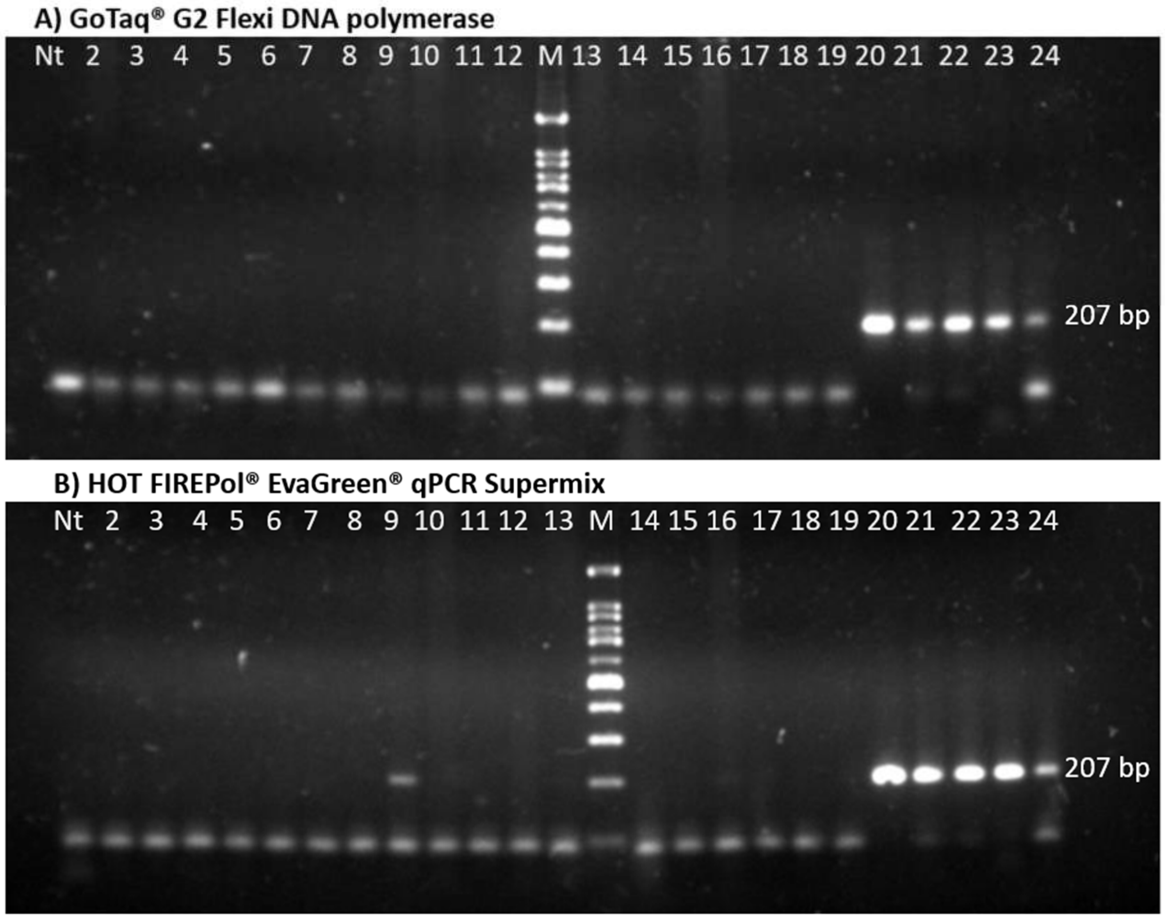

Figure S4: An example of specificity test with GoTaq® G2 Flexi DNA polymerase (Promega, USA) and HOT FIREPol® EvaGreen® qPCR Supermix (Solis BioDyne, Estonia) in end-point PCR.

Lines: Nt = no template, 2 = beef, 3 = maize, 4 = soya, 5 = sunflower, 6 = flax, 7 = tomato, 8 = potato, 9 = barley, 10 = wheat, 11 = rye, 12 = oat, 13 = pepper, 14 = rice, 15 = mustard, 16 = common poppy, 17 = oriental poppy, 18 = iceland poppy, 19 = tulip poppy, 20 = opium poppy (Aplaus), 21 = opium poppy (Maraton), 22 = opium poppy (Opal), 23 = opium poppy (Opex), 24 = poppy pie; M = 100 bp marker.

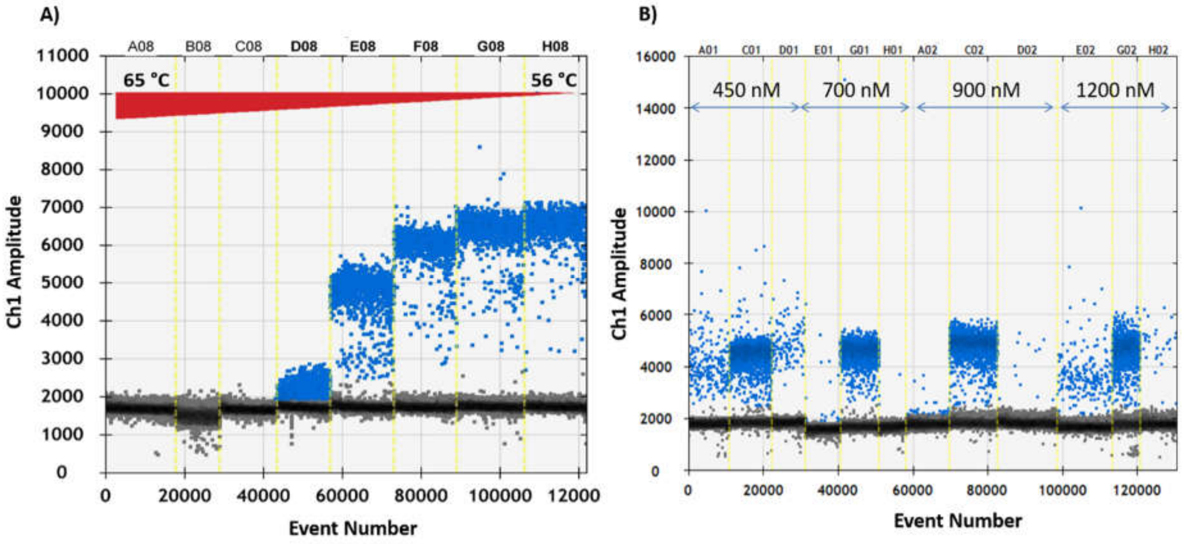

Figure S5: Optimization of ddPCR assay with COR primers: A) temperature gradient (65 – 56 °C) tested with *P. somniferum* L. (Opal) DNA; B) concentration of primers in the reaction (450 - 1200 nM), from left: no template control, *P. somniferum* L. (Opal), *P. orientale*; four times in a row. Optimization of reaction conditions provided a better distribution of clusters of negative and positive droplets, and thus also a difference in the calculated concentrations of targeted DNA in the sample. For example, the difference between the measured concentration at 56°C and 62°C was 16 copies per microliter.

Table S1: List of analysed samples from the Czech market network.

Product (producer/seller)	Declared contain of poppy*	Detection of opium poppy (COR)
Poppy seed cake I	13.50 %	+
Poppy seed cake II	N	-
Poppy seed cake III, cut	N	-
Poppy seed cake IV	N	+
Poppy seed cake V	N	+
Poppy seed cake VI	N	+
Strudel with poppy seed filling	16.12 %	+
Tip with poppy seed feeling	32.24 %	+
Czech blue poppy, seeds	100 % (*P. somniferum*)	+
Duets with poppy seed filling /poppy seed tip	10.85 %	+
Czech poppy seed buns I	11.52 %	+
Poppy seed buns II	18.00 %	+
Janek´s buns III	14.56 %	+
Poppy seed strudel II	14.82 %	+
Poppy seed filling	40 % (ground opium poppy)	+

* N = not declared. Only a few products stated the exact species of poppy used for production; if specification is not written, the producer declared only "poppy" (in general).
